# Vascular Embolism After Dull Needle Injection of Hyaluronic Acid Into Glabellar Wrinkles in Preformed Subdermal Tunnels: A Case Report and Review of the Literature

**DOI:** 10.1111/jocd.70314

**Published:** 2025-07-01

**Authors:** Tianqi Shi, Hui Lin, Jingjing Zhang, Chengjiang Wei, Yuanbin Ou, Linna Wang, Qing Wen

**Affiliations:** ^1^ Department of Laser and Plastic Surgery The First Affiliated Hospital of Guangxi University of Chinese Medicine Nanning Guangxi People's Republic of China; ^2^ School of Basic Medical Sciences Guangxi University of Chinese Medicine Nanning Guangxi, People’s Republic of China

**Keywords:** dull needle, glabellar wrinkles, hyaluronic acid, preformed subdermal tunnels, vascular embolism

## Abstract

**Background:**

More and more people inject fillers to fill their glabellar wrinkles, and complications are on the rise.

**Case Presentation:**

A 36‐year‐old woman was given a local infiltration anesthetic, and then a 22G sharp needle with an empty syringe was utilized to puncture several times near the subcutaneous tissue to form a tunnel. When we squeezed the tunnel, there was no bleeding, and then the needle was replaced by a 22G dull needle. The empty syringe was replaced by a hyaluronic acid gel syringe, and a small amount of hyaluronic acid was slowly given while withdrawing the needle. Skin swelling and bruising occurred 15 h after injection. Hyaluronase was injected into the ischemic area and treated with specific electromagnetic wave and vasodilators. Epidermal erosion appeared; however, it healed within 1 week without scar.

**Conclusion:**

Vascular embolism may be due to the needle piercing the blood vessel, a small amount of gel was forced into the pierced vessel, or the shear force of blood flow carried tiny gel particles into the vessel. The glabella is one of the most dangerous locations for hyaluronic acid injection because of possible vascular embolism. The use of preformed tunnels, thick dull needle, and aspiration without blood are not absolutely safe, and patients should be informed of the clinical manifestations of embolization before injection, and follow‐up informed after injection.

## 1 Introduction

For people who habitually frown, with the increase of age, the glabellar wrinkles will become deeper and deeper, and some people will still have them after stopping frowning. Glabellar wrinkles give people a frown, sadness, and aging appearance, which greatly affects the beauty of the face. Glabellar lines (12.4%) were the top research hotspots in the East Asian and Western regions [1].

For mild glabellar lines, Botulinum toxin type A(BoNT‐A) injection can achieve satisfactory results [2]. BoNT‐A was first isolated in 1946, and since then, several formulations have been developed and widely used to treat wrinkles by inducing muscle paralysis [3]. For patients with moderate to severe glabellar frown, the combined administration of BoNT‐A and hyaluronic acid filler could be a considerable treatment for improving wrinkles [4]. We used BoNT‐A injection combined with preformed subcutaneous tunnels and dull needle hyaluronic acid to remove glabellar wrinkles and achieved satisfactory results. But in September 2024, a rare case of vascular occlusion occurred. The early clinical manifestations, anatomical mechanism, possible etiology, prevention, and treatment measures of this case are analyzed in combination with relevant literature.

### 1.1 Case Presentation

A 36‐year‐old woman presented to the plastic surgery department of our hospital for glabellar wrinkles of 3 years. The patient complained that she had been injected with BoNT‐A at an outside medical facility 1 month ago for glabellar wrinkles, but still felt the depression existed, and wanted to fill it. The patient has no history of hyaluronic acid filler, no history of allergies, no autoimmune diseases, no hypertension, and no diabetes. Physical examination revealed static depression in the glabellar furrow, unable to wrinkle the brow with force, no scar, no redness and swelling or infection in local skin. Diagnosis: After BoNT‐A injection, the glabellar wrinkles are deeply sunken. Before surgery, patients were informed of the risk of HA injection, postoperative precautions and asked to sign an informed consent form.

#### 1.1.1 Injection Process

**FIGURE 1:**
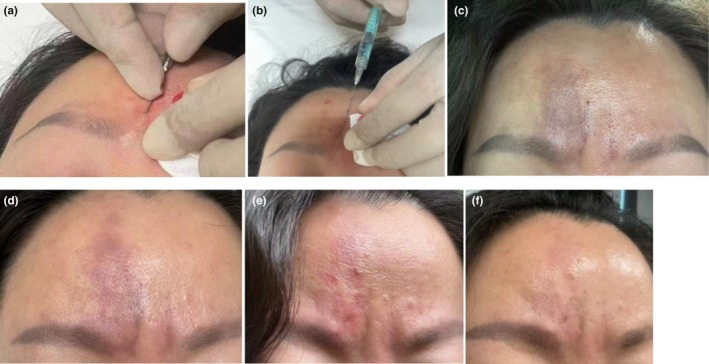
(a) Preformed subdermal tunnels, (b) withdraw the needle while slowly injecting a small amount of hyaluronic acid, (c) skin swelling and bruising 15 h after injection, (d) the swelling andbruising receded mostly after multiple injections of hyaluronidase, (e) on the second day, the bruise subsided and three areas of epidermal erosion, (f) healing within 1 week without scar.

The patient was injected with cross‐linked sodium hyaluronate gel (concentration of 22 mg/mL, YVOIRE volume s, LG Chem, Korea). The procedure was performed by a surgeon with clinical experience, and the depressed wrinkles were designed as a longitudinal axis to puncture the preformed tunnel. After iodine tincture disinfection, local infiltration anesthesia(2% lidocaine+l:200000 epinephrine) was successful, and then a 22G sharp needle with an empty syringe was used to puncture several times near the subcutaneous tissue to form a tunnel(Figure 1a). No bleeding was seen when squeezing the tunnel, and then the needle was changed to a 22G dull needle and no bleeding was seen when withdrawing. The empty syringe was changed to a hyaluronic acid gel syringe, and a small amount of hyaluronic acid was given slowly while withdrawing the needle (Figure 1b). The skin color was no different from before the injection, and there was no pain with 0.2 mL on the left and 0.25 mL on the right. After 30 min of ice pack, the patient left the hospital with no change in skin color and no pain or other discomfort. We told her to come to the hospital promptly if she has pain and (or) changes in skin color. Follow‐up at 8 pm reported no special condition; follow‐up at 8 am the next day reported that the skin on the right turned dark, and the patient was instructed to return to the hospital immediately. Inspection revealed ecchymosis of the skin in the area of the superior trochlear artery along the eyebrow on the right side, slight swelling, and local tenderness (Figure 1c). The patient had normal eye movements, normal visual examination, and good eyelid closure. Because of the high suspicion of vascular embolism, the patient was advised to dissolve with hyaluronidase, and agreed.

### 1.2 Embolization Treatment

Skin allergy tests were performed, and the results showed that hyaluronidase allergy was negative. Multiple injections of hyaluronidase (1500u/bottle, SPH No. 1 Biochemical & Pharmaceutical Co. Ltd.) were given in the ischemic area above the right eyebrow, with an injection depth of subcutaneous and muscular layers at multiple levels. The injection dose was 600 U of hyaluronidase, and a specific electromagnetic wave was given for 20 min. After half an hour of exposure to electromagnetic waves, the bruising has obviously subsided, and tenderness has been alleviated (Figure 1d). Patients took oral Aspirin tablets 0.3 g/day, once/day, for 5 days; Dexamethasone tablets 0.75 mg/time, 3 times/day, for 3 days; hot compress 3 times/day.

On the second day, the bruising was seen to be fading, there were three areas of superficial erosion, and the specific electromagnetic wave was to be irradiated for 20 min, and 2% mupirocin ointment and recombinant bovine basic fibroblast growth factor gel were applied externally (Figure 1c).

On the third day, the patient did not come over, complaining that the bruising had basically subsided and was instructed to apply mupirocin ointment and recombinant bovine basic fibroblast growth factor gel.

On the seventh day, she was followed up by telephone, complaining that the scab was falling off, the color was close to the skin, but the redness slightly more obvious after tension or exercise. (Figure 1F).

## 2 Discussion

The mechanism of glabellar wrinkles is due to excessive contraction of the glabellar complex, combined with long‐term frowning, skin aging, muscle contraction strength, and rich facial movements, all of which accelerate the formation of wrinkles. Honeck [5] divided glabellar wrinkles into 4 grades: grade 0, no wrinkle; grade 1, mild wrinkle; grade 2, moderate wrinkle; grade 3, severe wrinkle with local depression. Depending on the severity of glabellar wrinkles, the doctor can choose different treatment methods. Hyaluronan (HA)‐based soft‐tissue fillers are injectable crosslinked hydrogels aimed to counteract facial skin aging signs via minimally invasive procedures [6]. HA dermal fillers are generally safe and effective, with most adverse events being transient and mild to moderate in severity. Severe adverse events, although rare, do occur and are generally non‐treatment related [7, 8]. Vascular complications arising from dermal filler treatments pose significant risks, including ischemia, tissue necrosis, and severe outcomes like blindness and pulmonary embolism [9, 10].

There are 6 dangerous areas for injection on the face: glabella, temporal, infraorbital, nasolabial fold, lips, or sum of the nose, and once a vascular embolism occurs in these areas, there is no other collateral circulation to compensate. Injections in the periosteum layer at the glabellar region or sub‐superficial musculoaponeurotic system layer of the nasal dorsum and nasolabial fold are not advised [11]. In the eyebrow level, the epidermis‐artery distance (EAD) is between 1,8 and 5.9 mm, and the artery‐periost distance (APD) is between 0.7 and 3.7 mm. In the 1.5 cm superior level of the eyebrow, the EAD is between 1.8 and 5.1 mm and the APD is between 0.6 and 3.8 mm [12]. Safe filler injections to correct the glabellar frown lines can be possible with intradermal injections. de Maio M [13] believes that the supraorbital artery and the supratrochlear artery exit the orbit at a deeper position. If a sharp needle is at the periosteum, it is necessary to be 2 cm above the eyebrow. Among the 74 wrinkle lines, the supratrochlear arteries were located either at the glabellar wrinkle lines (30/74, 41%) or lateral to the glabellar wrinkle lines (44/74, 59%) [14]. Central (CAs), paracentral (PCAs), and reverse dorsal nasal arteries (rDNAs) were found in 70 (38.9%), 58 (32.2%), and 16 (8.9%) of the 180 hemifaces, respectively [15]. The average distance from midline to the central and PCA was 4.1 mm (range 1.8–6.7 mm) and 8.2 mm (6.8–10.1 mm), respectively. The average depth from the skin to the central and PCA was 2.7 mm (2.2–3.2) and 3.0 mm (2.6–3.4 mm), respectively [16]. Experts suggest that the forehead and glabella are not suitable for deep periosteum layer injection, and it is recommended to use superficial multi‐point injection to prevent damage to the superior trochlear artery and superior orbital artery, resulting in injection substance entering the blood vessels.

Static lines with local depression belong to severe glabellar lines, which are caused by long‐term muscle contraction, leading to loss of subcutaneous fat and formation of scar‐like tissue between the subcutaneous and muscle. The scar tissue has poor elasticity, and the scar can anchor the blood vessels in the tissue. When blunt needles encounter these scar‐fixed vessels, their ability to slide along the surface of the vessels is reduced. The above reasons will increase the risk of embolization [17, 18]. Be particularly careful if there is a scar at the injection site, as it can make the vessels fixed, making it easy to puncture the vessels, and the same true for moderate and severe glabellar lines.

Deep glabellar furrows are so deep that the filler may not be able to enter the depressed area but may be injected into the adjacent parts. Injection techniques like the fern leaf and duck walk methods are preferred to minimize the risk of vascular complications and achieve a smooth, natural appearance [19], but the effect is not good for deep glabellar wrinkles. We adopt the method of puncturing and stripping the depressed area, and then filling the preformed tunnel, which solves the problem of filler flowing to its adjacent parts. The preformed subcutaneous tunnel is widely used in fat transplantation, and it is proved to be a safe method to avoid vascular injury and embolism. In all cases, fat grafts were injected by blunt cannula using a tunneling technique in different planes [20, 21]. The sharp end of an 18‐gauge needle is then used to create a tunnel in the subcutaneous plane beneath each line while releasing any fibrous bands [22]. The preformed subdermal tunnels are a simple and reliable method to solve deep wrinkles or depressed scars.

To prevent vascular embolism, people try many methods, using blunt needles instead of sharp needles to prevent puncturing the blood vessels. Since the diameter of the key facial arteries such as the dorsal nasal artery, supratrochlear artery, and supraorbital artery is 1 mm, a large‐diameter blunt needle is safer than a small‐diameter blunt needle, and a large‐diameter blunt needle cannot penetrate the artery [23]. Cassiano D reported [24] a case of skin necrosis following the use of a 25G blunt needle, suggesting the use of a needle of 23G or larger for facial filler injection. This will minimize the risk of arterial injury and reduce the chance of vessel puncture.

Based on the anatomical consideration of the blood vessels of the forehead and the recommendation of the experts, the method we used was to design a pre‐planned tunnel the depressed wrinkle as the longitudinal axis. Most patients were satisfied, and no vascular embolism occurred. This case involved superior trochlear artery embolism, which may be due to the shallow position of the patient's artery, stabbed by a 22G injection needle or damaged during dissection. The diameter of the superior trochlear artery is about 1 mm, and the outer diameter of the 22G blunt needle is 0.7 mm. Although the blunt needle is smaller than the diameter of the superior trochlear artery, the possibility of the 22G blunt needle slowly entering the pre‐made tunnel and puncturing the blood vessel is very small. With such a thick needle puncturing the blood vessel, there should be blood in the aspiration and bleeding when the needle is withdrawn from the blood vessel; if the hyaluronic acid gel is mistakenly injected into the blood vessel cavity, it will also cause more serious symptoms of vascular lumen obstruction and a larger area of necrosis. We speculated that the blood vessel was punctured or peeled off, and the local tissue became edematous and pressure increased after hyaluronic acid absorbed water, and a small amount of gel squeezed into the broken blood vessel. The HA inside the artery may have traveled over time and reached a terminal distal branch, which generated localized skin damage and pain [25]. Another possibility was that a gel just plugged the punctured vessel, and the shear stress of the blood flow took the tiny gel particles into the vessel, where they were carried downstream to eventually plug the tiniest capillaries, thus causing the skin to slough off. Hyaluronic acid and hydroxylapatite fillers break up into small particles immediately after injection into a flowing system, generating emboli rather than a column of filler [26].

The typical manifestations of vascular embolism are severe sharp pain and pale, black and spotted skin color. In atypical vascular occlusion, there is no obvious change in skin color in the early stage [27], and the embolism can be judged by checking the capillary filling time. It is generally considered that a capillary refill time of 1–2 s is normal. Note that the contralateral site of the same part should examined and compared. The capillary refill time is prolonged in arterial embolism and shortened in venous embolism [28, 29]. If vascular embolism is suspected, hyaluronidase can be injected for differential diagnosis, and ultrasound or angiography can also be used for diagnosis. Early detection of hyaluronic acid embolization can improve the therapeutic effect, speed up the recovery and reduce the necrotic area. Looking back on this process, local bruising appeared 15 h after injection, which improved after injection of hyaluronidase, indicating that embolism occurred. However, complications may occur even in the most skilled hands [30]. At present, no matter what injection technology and method, there is no guarantee that there will be no vascular embolism. Because the number of complications increases with the number of people injected, doctors should identify them early, intervene in time and speed up recovery. When vascular embolism is suspected, doctors use high‐dose hyaluronidase [31]. When local administration is not good or important vascular embolism occurs, intra‐arterial injection of hyaluronidase can treat vascular embolism. Intra‐arterial thrombolytic therapy (IATT) is safe and effective in reversing hyaluronic acid [32, 33].

## 3 Conclusion

Injection in eyebrow striation area is a high‐risk area, and blunt needle injection in stripping prefabricated tunnel is not absolutely safe. Vascular embolism may be due to the puncture of a vessel by the needle, and a small amount of gel was forced into the punctured vessel or the stress of the blood flow carried minute gel particles into the vessel. Doctors must be cautious in the operation, inform the clinical manifestations of embolism before injection, and follow up after injection.

## Author Contributions

H.L., J.Z., T.S. treated and dealt with the complications that arose in the article case; C.W., Y.O. provided some possible reasons for the complications and searches for literature; T.S., H.L., L.W. wrote the paper; Q.W. participated in the recording and collection of pictures.

## Disclosure

No potential conflict of interest was reported by the authors. This study has referred to the Helsinki Declaration. This article does not contain any studies with human participants or animals performed by any of the authors. The photos can be used or released with the patient's consent.

## Conflicts of Interest

The authors declare no conflicts of interest.

## Data Availability

All relevant data are within the paper.
